# Changing the paradigm in the cancer war: a strategy for the healthy and proper care of the sick

**DOI:** 10.3332/ecancer.2013.ed24

**Published:** 2013-07-16

**Authors:** Eduardo Cazap

**Affiliations:** Immediate Past-President, Union for International Cancer Control (UICC), Geneva, Switzerland; Founding President, Latin-American and Caribbean Society of Medical Oncology (SLACOM), Buenos Aires, Argentina; Executive Committee, National Cancer Institute, Ministry of Health, Buenos Aires, Argentina

The War on Cancer refers to the effort to find a cure for cancer by increased research to improve the understanding of cancer biology and the development of more effective cancer treatments. The aim of this effort was to eradicate cancer as a major cause of death. The signing of the National Cancer Act of 1971 by then US President Richard Nixon is generally viewed as the beginning of the war on cancer, though it was not described as a “war” in the legislation itself [1].

When President Nixon signed the cancer act, I was a medical doctor just graduating from medical school in Argentina. At that time, when cancer was a disease mainly managed through diagnosis and treatment, surgeons were the backbone of the medical apparatus. Some patients were also treated by radiotherapists and the so called, at the time, “chemotherapists”. The understanding of cancer was limited, cell kinetics was emerging and the availability of drugs was restricted to cytotoxic agents and a few hormones.

The objective of the Cancer Act was to defeat cancer by the year 2000 and the concept in the 70s was that cancer was typically a fatal disease, most of the treatments were palliative and that curability, apart from in the early stages of the disease, was uncommon. The basic idea of the Cancer Act was to use treatment as the main weapon, and that was, in part, an incorrect concept.

My own medical education at the University of Buenos Aires, the best university in my country, was at that point almost completely therapeutically oriented with poor information about primary and secondary prevention, health care issues, public health, and other relevant aspects of cancer. Over the last 40 years, the scientific situation has changed dramatically. Genetic and molecular information has been discovered, new strategies for primary and secondary prevention are presently in general practice, cancer vaccines are now a reality, and more sophisticated medical interventions are delivered in-clinic, along with new concepts of palliation, support and end of life care.

The last four decades have seen more developments and discoveries in cancer that in all the previous years of human history. Regrettably, this enormous amount of scientific knowledge and progress is currently not available to the majority of the world’s population. Access to proper cancer control is relatively limited, only possible in a number of developed countries and pockets of high-income populations.

After President Nixon’s Cancer Act, another important political event in the war against cancer was the “Charter of Paris.” Organized by Professor David Khayat, the Charter was signed in 2000 in the Élysée Palace by the President of France, Jacques Chirac, with the support of UNESCO, together with 100 international leaders of government, patient advocacy, cancer research organizations and corporations from different countries [2]. I remember very well that our perception at that moment was that the signature of the Charter was an “internationalization” of the war against cancer, advancing a global commitment against the disease.

This document was one of the predecessors of the UICC’s World Cancer Declaration, which calls on the world to take immediate steps to reduce the global cancer burden by committing to its 11 Declaration targets, and providing resources and political backing for the priority actions needed to achieve them [3]. It is unacceptable today, in the year 2013, that all our scientific knowledge about cancer is available to but a small fraction of the world’s population, ostensibly 10 to 15%. What are the main causes for this intolerable situation?

There are several misconceptions, even at high medical and scientific levels. One frequent error is to consider cancer exclusively from the treatment perspective. We all know the key role of primary and secondary prevention, but this is not valid in many countries of the world. Only a few selected countries have established concrete strategies at national levels, with specific programs and policies for cancer prevention and early detection.

In recent years, the concept of Global Cancer Control has emerged. Cancer control encompasses a comprehensive number of actions from research, education, primary and secondary prevention to diagnosis, treatment, palliative care and end of life. Global Cancer Control entails global actions together with international collaborations, sometimes supported by International laws (such as the Framework Convention on Tobacco Control (FCTC)).

Another common fallacy is the belief that cancer is solely a medical problem. The extremely complex and multifaceted cancer crisis cannot be solved alone by the medical community and it is mandatory to consider a multi stakeholder approach.

Governments must assume responsibility, the private sector (an intrinsic component of the problem) must be a partner in the solutions, health care systems and care providers must be more efficient and trained, not only for treatment and palliation but, also, for public education and cancer prevention involving other sectors at different levels of health care systems.

We are now facing the next phase of the cancer war. The UN High-Level Summit on Non-Communicable Diseases (NCDs) was held in New York, September 19–20, 2010. After months of exacting negotiations in New York, UN Member States finally came to consensus on a Political Declaration, a major milestone in the history of cancer control. It builds on the World Health Assembly (WHA) resolution 58.22 on comprehensive cancer control and the World Cancer Declaration, demonstrating that world leaders have recognized the magnitude and impact of the cancer and NCD burden [4–5].

It also shows that world leaders now understand that the response to date has been inadequate in terms of resourcing and political commitment. With this Political Declaration, cancer is finally where it belongs – at the top of the global political agenda. This process has highlighted once again the enormous power of a united cancer control community, from the global to the local level, and working together using a common strategy.

Another point, generally unseen at the country governmental level, is that the cancer problem exceeds the capacity of a Minister of Health (MoH). Worldwide, specifically in developing countries, the MoH is commonly a weak position within the national government structure. Overburdened with political responsibilities, with a brief tenure in office, the MoH generally cannot and does not address the complexity of their country’s cancer problem.

It is critical to connect Ministers of Health with Ministries of Economy and Development to enable them to work together. It is highly recommended to create NCD’s Committees at governmental levels to work towards an integrated approach, not only with medical groups but also with governmental bodies, the private sector, academies, Universities, NGO’s and patient organizations.

What are my conclusions after more than 40 years since President´s Nixon National Cancer Act? What is the new paradigm in this enterprise? We need more and better public education about cancer. The public must be properly informed about the personal prevention of cancer. The media will also need to be educated and properly informed. We need more education of politicians about the looming disaster of cancer if governments don’t take urgent and proper actions. We need different doctors at all levels: generalists, primary care physicians, specialists and cancer experts. They will be aware and prepared to understand cancer and cancer control and Universities must update the medical curriculum following this concept. We urgently need doctors prepared to treat patients, but, more importantly, doctors need to be prepared to promote health. We need different health care systems, not only hospitals or cancer centres. We need hospitals going to the communities and participating in health promotion activities. We need cancer prevention programs integrated at the primary physician level to attend to the increasing needs of the healthy population. We need health care systems based upon access to care, efficiency, cost-benefit evaluations, quality-of-care and public health criteria, and integrated strategies.

In summary, global cancer control can be properly addressed if all participants understand that cancer is a problem of human development and that a horizontal strategy is mandatory. The cancer solution will be inevitably linked to the environment, science, economics, trade and regulations, together with proper research (epidemiologic, basic, translational, clinical and public) and scientific knowledge. In this scenario political will and international laws are a crucial component of this equation.

Ideally, the next objective from a global perspective will be to include cancer in the Millennium Development Goals, to promote and apply the World Cancer Declaration and the UN Political 2011 Declaration, to improve cancer research with a more open perspective, to make the cancer war a priority for all societies, and to re-engineer medical curriculums and health care systems in a new way, with doctors trained with a broad understanding of global cancer control.

For the common good, we should also continue the quest to improve society for a shared quality of life, with improved environments and promoting health at all levels to achieve good and efficient systems of public health care. Ultimately, we are all responsible for successful results in our collective War on Cancer. If each party clearly understands their vital role, we will advance in winning it.

“The inferior doctor treats actual sickness. The mediocre doctor attends to impending sickness; the superior doctor prevents sickness” Chinese proverb

The concepts expressed in this editorial are a personal view and must not be considered an official position of any of the organizations.

The author declares no conflict of interest.
